# 肺癌组织中DNA聚合酶β基因突变的研究

**DOI:** 10.3779/j.issn.1009-3419.2019.07.04

**Published:** 2019-07-20

**Authors:** 青峻 吴, 文鑫 田, 瀚博 于, 川 黄, 鹏 焦, 超 马, 永忠 王, 文 黄, 耀光 孙, 斌 艾, 宏峰 佟

**Affiliations:** 1 100730 北京，国家老年医学中心北京医院胸外科 Department of Thoracic Surgery, Beijing Hospital, National Center of Gerontology, Beijing 100730, China; 2 100730 北京，国家老年医学中心北京医院肿瘤内科 Department of Medical Oncology, Beijing Hospital, National Center of Gerontology, Beijing 100730, China

**Keywords:** DNA聚合酶β, 肺肿瘤, PCR扩增, 突变分析, DNA polymerase β, Lung neoplasms, PCR amplification, Mutation analysis

## Abstract

**背景与目的:**

DNA聚合酶β是参与DNA损伤修复的关键酶之一，国外有学者认为其编码基因*Polb*在30%的肿瘤中存在遗传突变，但受到所用标本量的限制，这一结论是否准确尚无定论。本研究基于基因测序技术，通过对69例肺癌患者组织标本的基因筛查，旨在明确DNA聚合酶β的基因突变在中国汉族人群肺癌患者中的发生频率。

**方法:**

利用盐析法提取69例肺癌患者的癌及癌旁组织基因组DNA，并用于扩增*Polb*基因全部14个外显子区及启动子区。通过与NCBI数据库中野生型基因序列进行比对，系统分析肺癌组织中的*Polb*基因突变及其频率。

**结果:**

相对于野生型，本研究共发现5种突变类型，其中3种（-196G > T, -188_-187insCGCCC, -168C > A）位于启动子区，2种（587C > G, 612A > T）位于外显子区，突变类型-188_-187insCGCCC和587C > G尚未见文献报道，后者可引起第196位氨基酸由苏氨酸突变为丝氨酸。另一方面，癌和癌旁组中均可检测到所有5种突变类型，提示这些突变并非肺癌组织所特有。

**结论:**

肺癌组织中未发现特异性表达的*Polb*基因突变位点，*Polb*基因突变可能不是中国汉族肺癌患者的肿瘤标志物。

## Abstract (en)

**Background and objective:**

DNA polymerase β is one of the key enzymes for DNA repair and it was reported that about 30 percent of different types of cancers carried mutations in its coding gene *Polb*. However, it is still controversial whether it is true or false because of the small sample size in these studies. In current study, we performed genetic screening of promoter and coding regions of *Polb* gene in 69 Chinese lung cancer patients using Sanger sequencing method, so as to elucidate real mutation frequency of *Polb* mutations in Chinese Han population.

**Methods:**

Salting out extraction method was used to get the genome DNAs from tumor and normal matched tissues of 69 lung cancer patients. The promoter and 14 coding regions of *Polb* gene were then amplified using these DNAs as the template. After purification, amplicons were sequenced and aligned to the wild type *Polb* gene in NCBI database, in order to find out the mutated sites of *Polb* gene in Chinese lung cancer patients.

**Results:**

In this study, we totally found only 5 mutated sites in *Polb* gene. In detail, 3 mutations (-196G > T, -188_-187insCGCCC, -168C > A) were located in the promoter region; 2 mutations (587C > G, 612A > T) were found in coding regions. Specially, mutations of -188_-187insCGCCC and 587C > G (resulting to the amino acid substitution of Thr to Ser at position 196) had never been reported by other groups before. However, all these 5 mutated sites could be detected in both tumor and matched normal tissues, which inferred that they are not lung tumor specific mutations.

**Conclusion:**

No lung tumor specific mutations of *Polb* gene could be found in Chinese lung cancer patients and *Polb* gene mutation might not be a molecular marker for Chinese lung cancer patients.

作为遗传信息的载体，基因组DNA很容易受到化学（如烷化剂）、物理（如紫外线）和生物作用（如活性氧）的损伤，这也是癌症成为现代社会人类生命首要威胁的原因之一。据报道，仅活性氧就可造成真核生物每个细胞、每天产生至少10, 000个DNA损伤位点^[1]^。碱基切除修复（base excision repair, BER）是细胞应对DNA损伤并自行修复的重要途径之一，DNA聚合酶β是参与BER过程最主要的DNA聚合酶，具有DNA聚合和dRP裂解酶双重活性^[2]^。国外已有研究^[3]^表明，约30%的常见肿瘤中存在DNA聚合酶β突变体，但受到所用标本量的限制（7种肿瘤仅149例病例），其结论是否准确尚待进一步证实。例如早期在结直肠癌中的研究仅有6例标本入组，有5例携带突变，突变率高达83%^[4]^，但随后较大样本的筛查表明，仅40%的肿瘤组织发现存在DNA聚合酶β突变体^[5]^。Bhattacharyya等^[6]^最早检测了肺癌肿瘤组织中DNA聚合酶β的突变情况，通过对11例肺癌患者癌组织和癌旁组织的RT-PCR检测，共发现了3种不同的变异体类型，但这些变异类型并不是肿瘤特异性表达的突变类型。目前还尚未见中国人群肺癌组织中DNA聚合酶β的突变情况的文献报道。本研究通过对69例肺癌患者肿瘤及癌旁组织开展DNA聚合酶β编码基因*Polb*的全外显子测序分析，系统研究了我国汉族肺癌患者中DNA聚合酶β的遗传突变规律。

## 1 材料与方法

### 1.1 样本基本信息

收集2018年2月-2018年9月北京医院住院收治的69例汉族肺癌患者的新鲜组织，术中取肿瘤组织及距肿瘤组织2 cm-3 cm处的癌旁组织，样本离体后5 min内分装至2 mL冻存管并迅速放至液氮中速冻，并置-80 ℃长期储存备用。入组样品均在术后均得到明确的病理学证实。所有纳入实验的研究对象均已签署知情同意书。患者中位年龄65.26岁（49岁-84岁），其中男性35例、女性34例，具体临床信息详见表 1。

**1:** 入组患者的临床病例特征 Clinical and pathologic characters of enrolled patients

Features	Number	%
Gender		
Male	35	51
Female	34	49
Age (yr)		
≥60	50	72
< 60	19	28
Smoking history		
Yes	19	28
No	50	72
Classification		
AC	57	83
SC	9	13
LLC	2	3
SCLC	1	1
pTNM stage		
Ⅰa1	2	3
Ⅰa2	24	35
Ⅰa3	16	23
Ⅰb	4	6
Ⅱa	3	4
Ⅱb	7	10
Ⅲa	11	16
Ⅲb	1	1
Carcinoma *in situ*	1	1
AC: adenocarcinoma; SC: squamous cell carcinoma; LLC: large cell lung cancer; SCLC: small cell lung cancer; pTNM: pathologic tumor-node-metastasis.		

### 1.2 基因组DNA提取及基因扩增

取适量癌及癌旁组织液氮下研磨成粉末，采用盐析法^[7]^提取基因组DNA。配制30 μL PCR反应混合物：含15 μL 2×Taq Plus Master Mix Ⅱ（南京诺唯赞生物技术公司），0.2 μmol/L扩增引物及50 ng提取的DNA。利用表 2中列出的引物及扩增条件进行如下扩增反应：94 ℃预变性3 min；94 ℃变性15 s，60 ℃-63 ℃退火15 s，72 ℃延伸30 s-3 min 45 s，共进行30个循环。扩增产物经2%琼脂糖凝胶电泳分离后，使用凝胶回收试剂盒（北京博迈德生物技术有限公司）回收目的条带，并送北京天一辉远生物技术有限公司，以表 3中列出的引物进行DNA Sanger测序。

**2:** *Polb*基因PCR扩增用引物 Primers used for amplification of human *Polb* gene

Region	Primer	Sequence (5′ -3′ )	Product (bp)	Annealing	Extension
Promoter+exon 1-2	polb-PE12-F1	GGAAACACAATCACCACAACCTT	1, 739	63 ℃	2 min
	polb-PE12-R1	ACCAGCCTCGATTCTTGCTTT			
Exon 3	polb-E3-F2	GCCTTGATGGATTTCTAATTGGTTT	449	63 ℃	30 s
	polb-E3-R2	GGACCAGATATGCTAGTGCCA			
Exon 4-5	polb-E45-F1	CGGGAGAATTTATTTTCACTGGGG	1, 331	63 ℃	1 min 30 s
	polb-E45-R1	TGGCTAAGGTACAGAGGTGGT			
Exon 6-7	polb-E67-F1	AGCACCCAGGAAAGTATCTGAC	3, 679	63 ℃	3 min 45 s
	polb-E67-R1	GAGCATGAAATAAACACCCGC			
Exon 8-9	polb-E89-F1	TGATCTGCTGGTATGGCACG	621	63 ℃	45 s
	polb-E89-R1	ATCATCCAGCCAAAAGGCCA			
Exon 10-11	polb-E101-F5	GTGTGTCATCAGCTTGGTTCC	1, 542	60 ℃	1 min 30 s
	polb-E101-R5	TGGCTACTGAACAGTCTCCAAG			
Exon 12-14	polb-E124-F1	TGTCTGTGTTTCGTGTTCAGG	3, 019	60 ℃	3 min
	polb-E124-R1	TCAGTAAGTAGGGGTAATGTGGTTT			

**3:** *Polb*基因启动子区及外显子测序引物 Sequencing primers for promoter and exons of *Polb* gene

Region	Primer	Sequence (5′ -3′ )
Primer+exon 1	polb-PE12-F3	GAACCCAGGAGTTACGCT
Exon 2	polb-PE12-R1	ACCAGCCTCGATTCTTGCTTT
Exon 3	polb-E3-R2	GGACCAGATATGCTAGTGCCA
Exon 4	polb-E45-F1	CGGGAGAATTTATTTTCACTGGGG
Exon 5	polb-E45-R1	TGGCTAAGGTACAGAGGTGGT
Exon 6	polb-E67-F1	AGCACCCAGGAAAGTATCTGAC
Exon 7	polb-E67-R2	CCCGCTGACTGCCTGATACA
Exon 8-9	polb-E89-R2	CAGCCAAAAGGCCAAAGACAA
Exon 10	polb E10-SER2	TAGACTGTCCTCCCAGCA
Exon 11	polb E11-SEF2	TACTCTGGATGCTGAGGTGGGA
Exon 12	polb-e12-seF	GGTAGGGATAGTGTATTGCTC
Exon 13	polb-e123-seqR	GTTGCCTGAAGGACTAAAA
Exon 14	polb-E124-R2	CAGTAAGTAGGGGTAATGTGGTT

### 1.3 基因测序结果分析

NCBI数据库中下载野生型人*Polb*基因的DNA序列为标准序列（NC_000008.11），使用Lasergene软件（版本号7.1）中的Seqman组件对获得的测序峰图文件进行比对拼接，杂合子检测阈值设置为 > 15%，并人工对软件检测出的候选杂合子利用Chromas软件（版本号2.22）进行逐个鉴定，以排除测序噪音对研究结果的干扰。为保证研究结果的可信性，利用与原测序引物相反方向的PCR扩增引物进行反向测序以二次验证其真伪。

## 2 结果

### 2.1 *Polb*基因的PCR扩增

利用表 2中列出的引物及扩增条件，经琼脂糖凝胶电泳检测，本研究成功获得了与预期条带大小完全相符的扩增产物（图 1），Sanger测序分析表明，各扩增产物均来自于人*Polb*基因。

**1:**
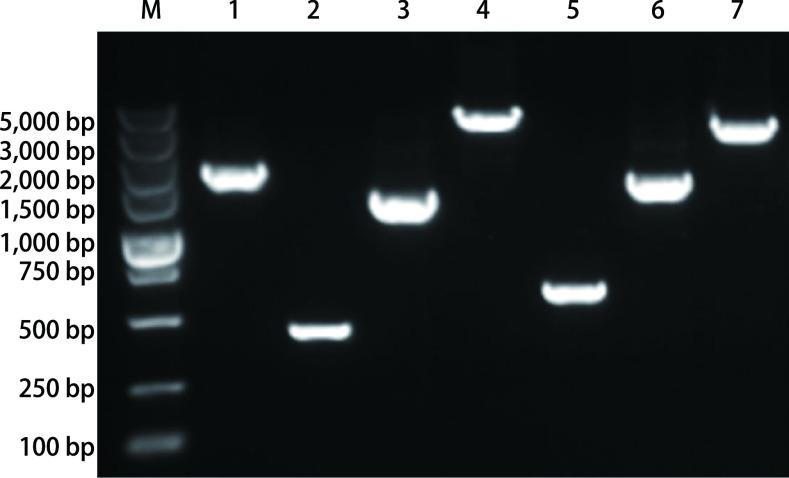
*Polb*基因PCR扩增产物电泳图谱 PCR products of *Polb* gene in lung cancer patients. M: DL5000 marker, 1-7: PCR products corresponding to the column of regions in Tab 2.

### 2.2 肺癌组织中*Polb*基因的突变频率分析

本研究系统分析了69例肺癌患者癌组织及癌旁组织中*Polb*基因的突变情况，经与野生型序列的比对分析，共计发现5种不同的突变类型，其中3种突变（-196G > T, -188_-187insCGCCC, -168C > A）位于启动子区，2种突变（587C > G, 612A > T）位于编码区，587C > G突变可引起第196位氨基酸由苏氨酸替换为丝氨酸，612A > T突变为同义突变（图 2）。各突变体的基因频率及基因型频率详见表 4。所有这些突变位置均可在肿瘤及对应的癌旁组织中同时被检测到，表明这些*Polb*基因的突变类型并非肺癌组织所特有。

**2:**
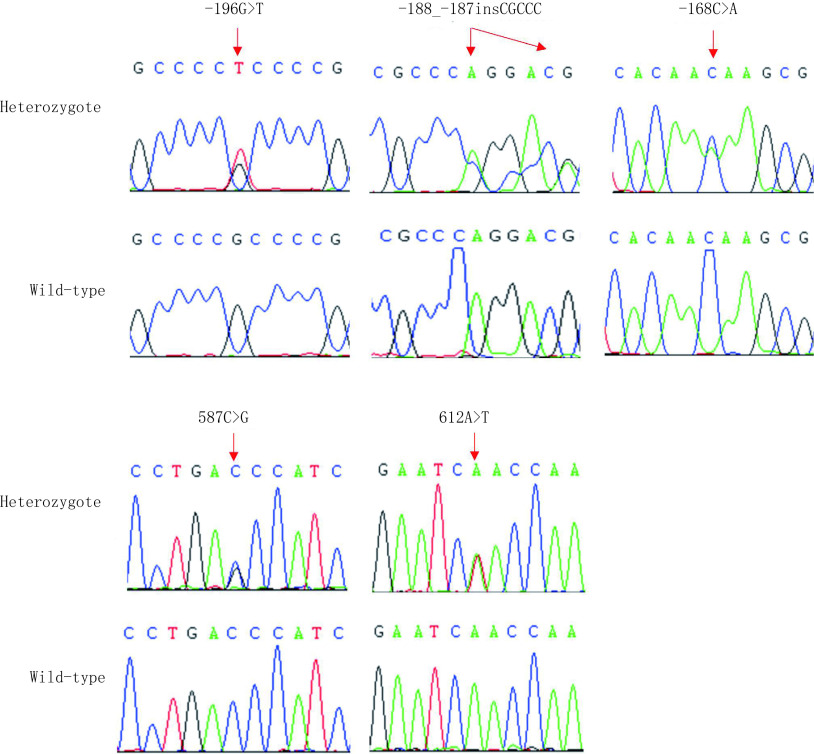
*Polb*突变体的典型测序图谱 Typical sequencing electropherogram of *Polb* mutations

**4:** 肺癌患者中发现的*Polb*基因突变类型及分布频率 Mutations and their frequencies of *Polb* gene in lung cancer patients

Mutation	Allele frequency (*n*=138)	Genotype frequency (*n*=69)
-196G > T	7.25%	13.04%
-188_-187insCGCCC	1.45%	2.90%
-168C > A	2.17%	4.35%
587C > G (resulting to T196S)	1.45%	2.90%
612A > T (resulting to S204S)	3.62%	7.25%

## 3 讨论

DNA聚合酶是碱基切除修复中最关键的聚合酶，早期的遗传筛查研究^[3, 8, 9]^表明，肺癌、胃癌、乳腺癌、肠癌、前列腺癌肿瘤组织中均可检测到*Polb*基因变异体的高表达。随着研究的深入，部分学者认为大多数肿瘤中均存在*Polb*基因的突变，其中包括大片段的缺失、插入及各种形式的点突变，突变频率从15%-75%不等，平均频率为30%左右，且多数突变体均可导致DNA聚合酶β活性下降，进而导致基因组不稳定性增加及细胞癌化^[3, 10]^。但另一部分学者^[11-13]^认为，这些突变绝大多数只发生在RNA水平，是由于RNA的选择性剪切导致，而非基因组DNA发生突变所致，其中外显子2和外显子11的缺失突变类型发生频率最高。

Bhattacharyya等^[6]^最早在肺癌肿瘤组织中检测到了DNA聚合酶β，11例肺癌肿瘤组织中检测到了3种DNA聚合酶β变异体，分别是87 bp、140 bp的缺失和105 bp的插入。这些变异体均只在cDNA中检出，基因组DNA中未检出，且癌旁组织和正常人的肺组织标本也存在同样的突变。从基因结构分析，这些突变体应该分别来自外显子11的缺失、外显子13的缺失和外显子α的插入，是由于RNA的选择性剪切所致，而非来源于基因组DNA的突变。本研究通过对69例汉族肺癌患者组织标本的基因筛查，基因组DNA中未发现肿瘤特异性表达的*Polb*基因突变。与Bhattacharyya等^[6]^的研究类似，所有发现的5种突变在癌和癌旁组织中均可被检出，提示这些*Polb*基因突变并不是肺癌肿瘤组织所特有。

本研究发现的*Polb*基因突变类型有2种发生在编码区，其中587C > G可引起氨基酸水平上第196位由苏氨酸T突变为丝氨酸S，未见有相关文献报道，可能是汉族人群特有的*Polb*基因突变类型，在本研究入组样本中有2.9%的人群携带此突变。196位苏氨酸位于DNA聚合酶β的C结构域，是发挥DNA聚合酶活性的重要区域^[14]^，该突变是否会引起DNA聚合酶β功能发生改变不得而知，需要开展体外蛋白活性实验及体内细胞转化实验加以研究。

本研究在*Polb*基因启动子区共检测到3个突变位点，其中-188_-187insCGCCC突变类型为首次报道。如图 3所示，3种检测到的突变体均位于*Polb*基因核心启动子区内^[15]^，位于或紧邻转录因子结合的核心区段。-196G > T和-168C > A由郑州大学的董子明组在食管癌组织中被首次报道^[16]^。体外实验^[17]^表明，两种突变均可引起*Polb*基因启动子活性的明显增强，并导致细胞对化疗药cisplatin的抗药性^[18]^。本研究在启动子区发现了一种新的插入性突变体-188_-187insCGCCC，其插入位点位于转录因子SP1的核心结合位点，且插入碱基多达5 nt，推测该插入突变可能会显著降低对转录因子SP1的亲和力，进而降低启动子的转录启动活性，并导致DNA聚合酶β蛋白表达量的降低。

**3:**
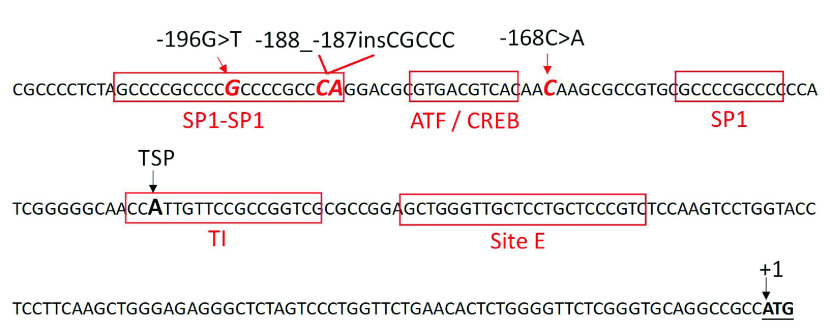
3种突变体在*Polb*基因核心启动子区的位置示意图 Site location of three detected mutations in human *Polb* core promoter. The mutated sites are indicated as red bold italics and sequences matching SP1 and ATF/CREB-binding sites are boxed. The nucleotide A in start codon is designated as +1. TSP: transcription start point; TI: the 18-nt tsp region.

总之，本研究通过对69例汉族肺癌患者肿瘤组织的基因筛查，共发现5种*Polb*基因的突变类型，其中有两种突变类型为首次报道。考虑到发现的突变位点的种类和发生频率较已有西方人群的报道明显偏少，且这些突变点在癌和癌旁组织中均可被检出，提示这些突变并不是肺癌肿瘤组织所特有，*Polb*基因突变可能不是中国汉族肺癌患者的肿瘤标志物。
